# The Use of Twitter to Track Levels of Disease Activity and Public Concern in the U.S. during the Influenza A H1N1 Pandemic

**DOI:** 10.1371/journal.pone.0019467

**Published:** 2011-05-04

**Authors:** Alessio Signorini, Alberto Maria Segre, Philip M. Polgreen

**Affiliations:** 1 Department of Computer Science, University of Iowa, Iowa City, Iowa, United States of America; 2 Department of Epidemiology, College of Public Health, University of Iowa, Iowa City, Iowa, United States of America; 3 Department of Internal Medicine, Carver College of Medicine, University of Iowa, Iowa City, Iowa, United States of America; Yale University, United States of America

## Abstract

Twitter is a free social networking and micro-blogging service that enables its millions of users to send and read each other's “tweets,” or short, 140-character messages. The service has more than 190 million registered users and processes about 55 million tweets per day. Useful information about news and geopolitical events lies embedded in the Twitter stream, which embodies, in the aggregate, Twitter users' perspectives and reactions to current events. By virtue of sheer volume, content embedded in the Twitter stream may be useful for tracking or even forecasting behavior if it can be extracted in an efficient manner. In this study, we examine the use of information embedded in the Twitter stream to (1) track rapidly-evolving public sentiment with respect to H1N1 or swine flu, and (2) track and measure actual disease activity. We also show that Twitter can be used as a measure of public interest or concern about health-related events. Our results show that estimates of influenza-like illness derived from Twitter chatter accurately track reported disease levels.

## Introduction

An estimated 113 million people in the United States use the Internet to find health-related information [1] with up to 8 million people searching for health-related information on a typical day. Given these volumes, patterns showing how and when people use the internet may provide early clues about future health concerns and/or expectations. For example, in the case of influenza, search engine query data from Yahoo [2] and Google [3] are known to be closely associated with seasonal influenza activity, and to a limited extent, actually provide some information about seasonal disease trends that precede official reports of disease activity.

Search query data provides one view of internet activity (i.e., the proportion of individuals searching for a particular topic over time), albeit one that is both noisy and coarse. The general idea is that increasing search query activity approximates increasing interest in a given health topic. Since some search query data also carries geographic information (generally based on the issuing IP address), it may also be possible to detect simple geospatial patterns. But search query data do not provide any contextual information; questions like why the search was initiated in the first place are difficult to answer. People search for health information for any number of reasons: concern about themselves, their family or their friends. Some searches are simply due to general interest, perhaps instigated by a news report or a recent scientific publication. Without sufficient contextual information, the relation between search query activity and underlying disease trends remains somewhat unclear.

Twitter is a free social networking and micro-blogging service that enables its millions of users to send and read each other's “tweets,” or short messages limited to 140 characters. Users determine whether their tweets can be read by the general public or are restricted to preselected “followers.” The service has more than 190 million registered users and processes about 55 million tweets per day [4]. A recent analysis of the “Twitter stream” revealed that a substantial proportion of tweets contain general chatter, user-to-user conversations only of interest to the parties involved, links to interesting pieces of news content, or spam and self promotion [5]. Despite the high level of noise, the Twitter stream does contain useful information. Many recent news events have been documented via Twitter directly from users at the site in real time: examples include US Airways flight 1549 landing in the Hudson River [6], or street riots during Iran's 2009 presidential elections. Because tweets are often sent from handheld platforms on location, they convey more immediacy than other social networking systems.

These examples suggest that useful information about news and geopolitical events lies embedded in the Twitter stream. Although the Twitter stream contains much useless chatter, by virtue of the sheer number of tweets, it will still contain enough useful information for tracking or even forecasting behavior when extracted in an appropriate manner. For example, Twitter data has been used to measure political opinion, to measure public anxiety related to stock market prices [7], national sentiment (i.e., happiness) [8], and to monitor the impact of earthquake effects [9]. In this study, we examine the use of information embedded in the Twitter stream to (1) track rapidly-evolving public sentiment with respect to H1N1 or swine flu, and (2) track and measure actual disease activity.

## Methods

### Twitter Data

**Figure 1:**
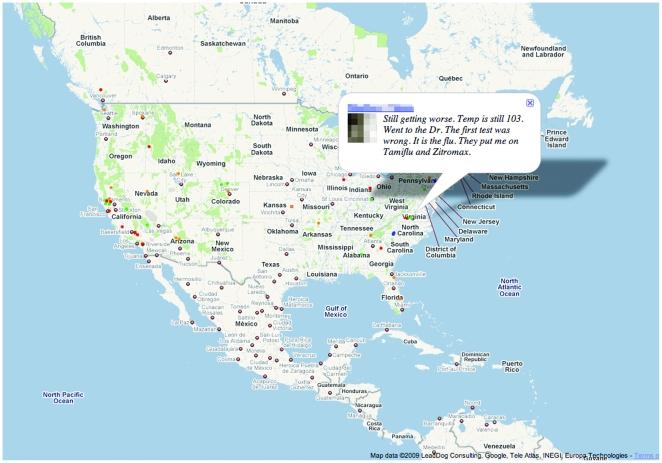
Influenza-Related Twitter Map. Color-coded dots represent tweets issued by users (shown at the users' self-declared home location). Hovering over the dot displays the content of the tweet; here, the user name is intentionally obscured. A client-side JavaScript application updates the map in near-real time, showing the 500 most recent tweets matching the preselected influenza-related keywords.

In order to explore public concerns regarding rapidly evolving H1N1 activity, we collected and stored a large sample of public tweets beginning April 29, 2009 that matched a set of pre-specified search terms: *flu, swine, influenza, vaccine, tamiflu, oseltamivir, zanamivir, relenza, amantadine, rimantadine, pneumonia, h1n1, symptom, syndrome,* and *illness*. Additional keywords were used to examine other aspects of public concern, including disease transmission in particular social contexts (i.e., keywords *travel, trip, flight, fly, cruise* and *ship*), disease countermeasures (i.e., keywords *wash, hand, hygiene* and *mask*), and consumer concerns about pork consumption (i.e., keywords *pork* and *bacon*). Each tweet is time-stamped and geolocated using the author's self-declared home location. A client-side JavaScript application was created to display a continuously-updated Google map with the 500 most recently matched tweets, yielding a real-time view of flu-related public sentiment in geographic context. Anyone visiting the web site could read any tweet by placing the cursor over its corresponding color-coded (by search terms) dot on the map (Figure 1).

Beginning on October 1, 2009, we collected an expanded sample of tweets using Twitter's new streaming application programmer's interface (API) [10] with the intent of estimating influenza activity. In addition, following discussions with public health officials, new search terms were added to investigate concerns about vaccine side effects and/or vaccine shortages: *guillain, barré, barre, shortage, hospital*, and *infection*.

Note that the Twitter stream is filtered in accordance with Twitter's API documentation; hence the tweets analyzed here still constitute a representative subset of the stream as opposed to the entire stream.

Moreover, because our main interest was to monitor influenza-related traffic within the United States, we also excluded all tweets tagged as originating outside the U.S., tweets from users with a non-U.S. timezone, and any tweets not written in English. We also excluded all tweets having less than 5 characters, those containing non-ASCII characters, and tweets sent by a client identifying itself as “API” (the latter are usually generated by computer and therefore tend to be “spam”). The remaining tweets were used to produce a dictionary of English words, from which all commonly-used keywords comprising Twitter's informal messaging conventions (e.g., #hashtag, @user, RT, links, etc.) were removed. Porter's Stemming Algorithm [11] was used to reduce inflected words to their root forms (e.g., “knowing” becomes “know”) in order to compress the size of the dictionary. We then compiled daily and weekly usage statistics for each dictionary term (i.e., number of tweets in which each term occurred), both nationally (by aggregating data for all valid locations) and at the CDC's influenza reporting region level [12]. Finally, because the volume of posts on Twitter varies over time as well as across geographic regions, usage statistics were expressed in terms of the fraction of the total tweets emitted within the corresponding time interval and geographic region.

### Influenza Data

Although influenza is not a nationally notifiable disease in the U.S., an influenza surveillance program does exist [13]. One component of this surveillance program is tracking reported influenza-like illness (ILI) during influenza season (usually October through May), since earlier detection can improve both clinical and public health responses. Members of the Influenza Sentinel Provider Surveillance Network report the total number of patients seen along with the number with ILIs (i.e., body temperature of 37.8°C or greater, cough and/or sore throat without any other apparent cause). Because ILI data are not uniformly reported by each state, the data are aggregated within the 10 CDC Influenza Reporting Regions [12] and subsequently weighted by regional population.

### ILI Estimation Model

The weekly term-usage statistics described previously were used to estimate weekly ILI. To determine the relative contribution of each influenza-related Twitter term, we used Support Vector Regression [14], an instance of the more general class of Support Vector Machines (SVM) [15], a supervised learning method generally applied to solve classification problems [16].

A classification system categorizes examples as instances of some class or concept. For example, one might build a classification system to discriminate between low and high risk for hospital readmission on the basis of information provided in a patient record. A learning method attempts to automatically construct a classification system from a collection, or training set, of input examples. Elements of the training set are usually represented as a collection of values for prespecified features or attributes; for this example, these features could be such measurable properties as age, recent hospitalizations, recent clinic visits, etc. Training set elements are marked a priori with their outcome, or class membership (e.g., “high risk”). Once generated, the classification system can then be used to predict the outcome of future examples on the basis of their respective feature values. Commonly-used learning methods include neural networks, Bayesian classifiers, nearest-neighbor methods, and so on; here, we use SVMs.

SVMs use quadratic programming, a numerical optimization technique, to calculate a *maximum-margin separator*, the hyperplane that maximally separates data points belonging to different classes in the multidimensional feature space, while tolerating only a prespecified error rate. Since the data are often not linearly separable (e.g., there is no simple linear expression, or hyperplane, that separates high risk from low risk of hospital readmission), a *kernel function* is used to project the data into a higher-dimensional space. If the new space has sufficiently high dimension, it ensures that a maximum-margin separating hyperplane exists and will be found efficiently even if the original data are not linearly separable. Commonly-used kernels include the radial basis function, hyperbolic tangent function, and the polynomial kernel function (used in this application).

When used for regression, SVMs produce a nonlinear model that minimizes a preselected linear-error-cost function where features serve as regression variables. Each input data point (or tweet) is described as a collection of values for a known set of variables or features: here, the feature set is defined as the collection of terms in the dictionary appearing more than 10 times per week. For each time interval, the value of a feature is given by its usage statistic for the corresponding term. Thus each tweet is encoded as a feature vector of length equal to the number of dictionary terms occurring more than 10 times per week, where the value assigned is the fraction of total tweets in that time interval that contain the corresponding dictionary term after stemming.

For the estimation work reported here, we relied on the widely adopted open-source libSVM implementation [17]. We trained our prediction models on weekly term-frequency statistics, using the ILI values reported by the CDC as a target for the weeks 2009/40 (October 4–10, 2009) through 2010/20 (May 16–22, 2010). Finally, as described in more detail below, we performed an out-of-sample validation using our data collected outside of CDC Region 2 (New York and New Jersey) to build the model used to estimate ILI in Region 2 based on Region 2 tweets.

## Results

### Tracking Public Interest with Twitter Data

**Figure 2:**
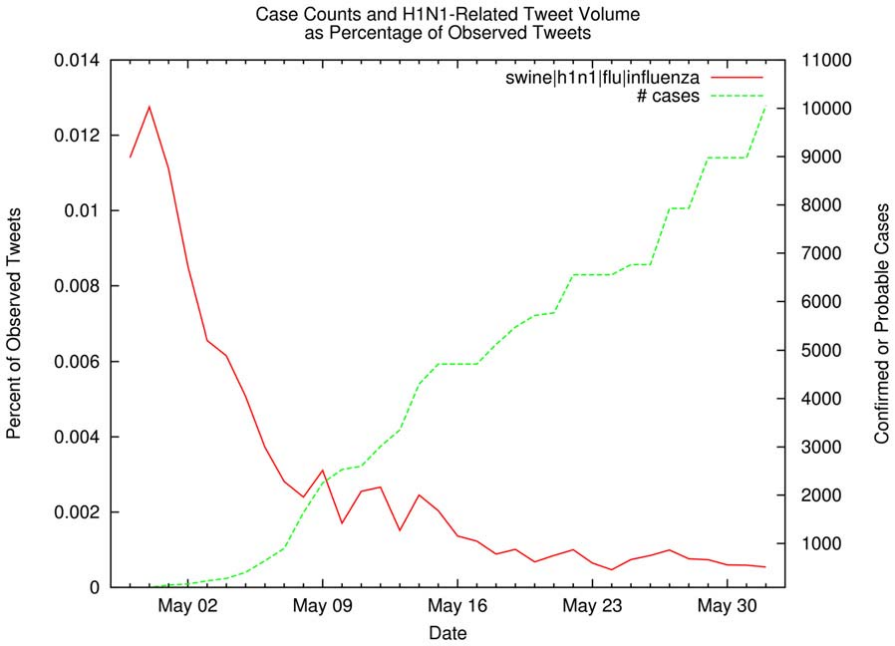
Case Counts and H1N1-Related Tweet Volume as Percentage of Observed Tweet Volume. The red line represents the number of H1N1-related tweets (i.e., containing keywords *swine, flu, influenza,* or *h1n1)* as a percentage of the observed daily tweets, while the green line shows the number of confirmed or probable H1N1 cases. Note that the volume of tweets pertaining to influenza steadily declined even though the number of cases continued to grow, reflecting a lessening of public concern about the severity of the pandemic.

**Figure 3:**
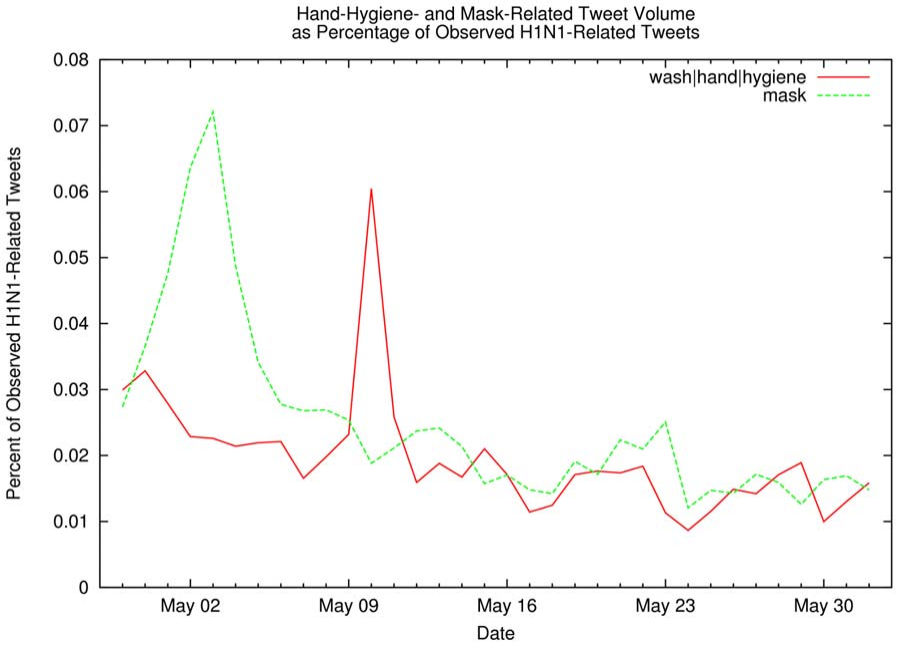
Hand-Hygiene- and Mask-Related Tweet Volume as Percentage of Observed H1N1-Related Tweets. Percentage of observed influenza-related tweets that also contain hand-hygiene-related keywords (red line) or mask-related keywords (green line). Spikes correspond to increased interest in these particular disease countermeasures, perhaps in reaction to, e.g., a report in the popular media.

**Figure 4 pone-0019467-g004:**
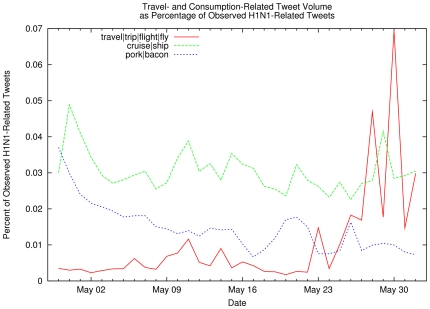
Travel- and Consumption-Related Tweet Volume as Percentage of Observed H1N1-Related Tweets. Percentage of observed influenza-related tweets that also contain travel-related keywords (red and green lines) or pork consumption-related keywords (blue line). The relative rate of public concern about pork consumption fell steadily during the month of May, in contrast to increased public concerns about travel-related disease transmission.

**Figure 5:**
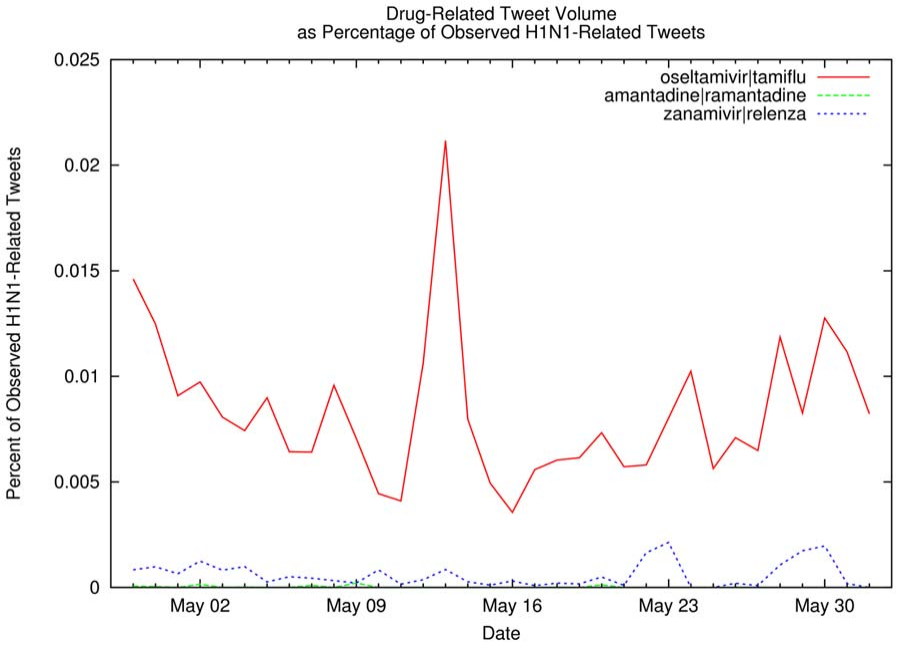
Drug-Related Tweet Volume as Percentage of Observed H1N1-Related Tweets. Percentage of observed influenza-related tweets containing references to specific anti-viral drugs.

The first data set consists of 951,697 tweets containing key words *h1n1, swine, flu* or *influenza* selected from the 334,840,972 tweets observed between April 29 and June 1, 2009 (because the size of the daily sample fluctuates, all results are reported as a percentage of observed tweets). These influenza-related tweets represent at most just over 1% of the sample tweet volume, and this percentage declined rapidly over time even as the number of reported H1N1 cases continued to climb (see Figure 2). Within the H1N1 tweet subset, we also counted tweets containing other influenza-related terms in order to study the evolution of public concerns about countermeasures (Figure 3 shows the percentage of influenza-related tweets that also mention hand hygiene or protective face masks), travel-related social contacts and consumption-related concerns (Figure 4 shows the percentage of influenza-related tweets that also mention airline trips, cruises, or pork products, like bacon), and treatment-related terms (Figure 5 shows the percentage of influenza-related tweets that also mention antiviral medications used to treat influenza). Note that each plot represents a rate within the rapidly declining ILI-related tweet volume sampled during the month of May, and not an absolute tweet count; as noted previously, since the Twitter sampling rate is known to fluctuate, percentages of observed volume are more representative than raw tweet counts. Since our intent was to track public interest as opposed to estimating disease activity, the periodic “spikes” of differing intensity observed in Figures 3 to [Fig pone-0019467-g004] 5 are not necessarily indicative of intense public interest, which one might expect to be manifested by a sustained elevated signal rather than sporadic short-lived bursts of tweets.

**Figure 6:**
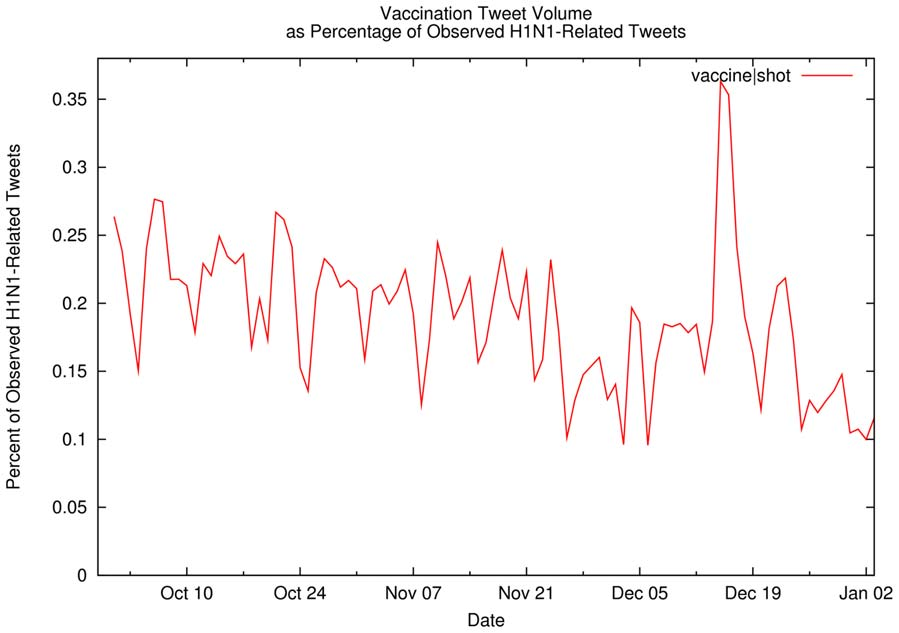
Vaccination Tweet Volume as Percentage of Observed H1N1-Related Tweets. Percentage of observed influenza-related tweets containing vaccination-related terms.

**Figure 7 pone-0019467-g007:**
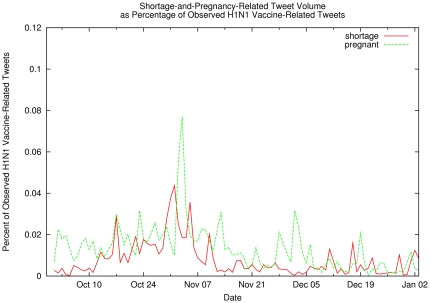
Shortage-and-Pregnancy-Related Tweet Volume as Percentage of Observed H1N1 Vaccination-Related Tweets. Percentage of observed H1N1 vaccination-related tweets containing terms related to pregnancy (green line) or vaccine shortage (red line). The relatively low rates observed may indicate either a lack of public concern or a lack of public awareness.

**Figure 8:**
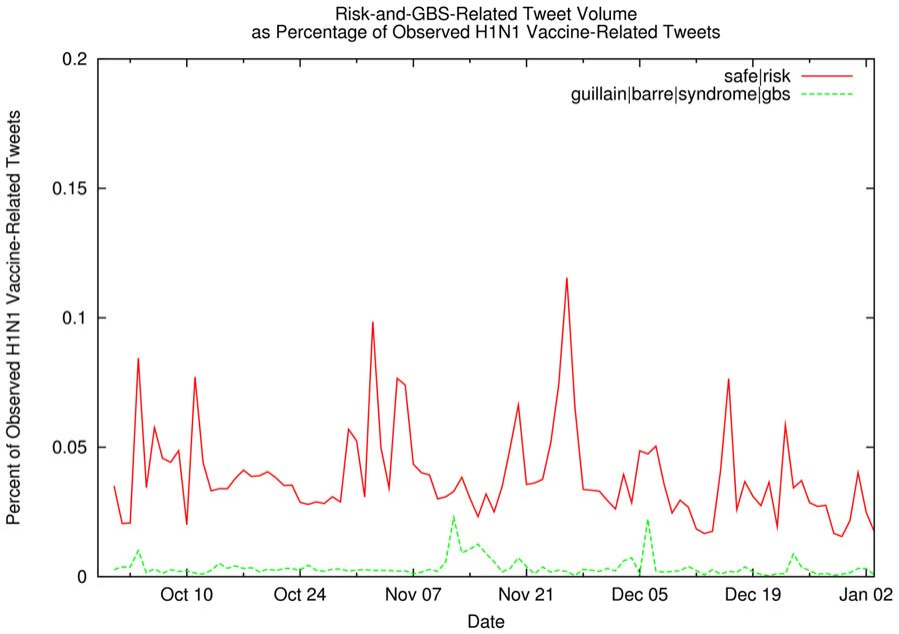
Risk-and-GBS-Related Tweet Volume as Percentage of Observed H1N1 Vaccination-Related Tweets. Percentage of observed H1N1 vaccination-related tweets containing terms related to risk perception (red line) or Guillain–Barré syndrome (green line).

The second data set consists of 4,199,166 tweets selected from the roughly 8 million influenza-related tweets (i.e., key words *h1n1, swine, flu* or *influenza)* observed between October 1, 2009 and the end of the year. Note that we excluded the approximately 4 million tweets that originated outside the U.S. or were determined to be “spam” by the method described previously. Using a temporally-specified subset of these data (i.e., all influenza-related tweets observed between October 1 and December 31), Figure 6 shows the percentage of influenza-related tweets that also mention vaccination concerns (i.e., key words *vaccine* or *shot*), while Figure 7 shows the percentage of vaccination-related tweets that also mention shortage- and pregnancy-related concerns (i.e., key words *shortage*, in red, or *pregnant,* in green). Similarly, since concerns about vaccine side effects may also affect vaccination uptake, Figure 8 shows the percentage of vaccination-related tweets that also mention side effects such Guillain–Barré syndrome, (in green: key words *guillain*, *barre*, *syndrome* or *gbs*) or the risks of vaccination (in red: key words *safe* or *risk*). While these search terms were selected to reflect vaccination uptake concerns within the public-health community, for the most part, simple inspection of Figures 6–8 does not reveal any evidence of sustained interest in vaccine-related issues within the Twitter community.

### Using Twitter to Make Real-Time Estimates of National Weekly ILI Levels

**Figure 9:**
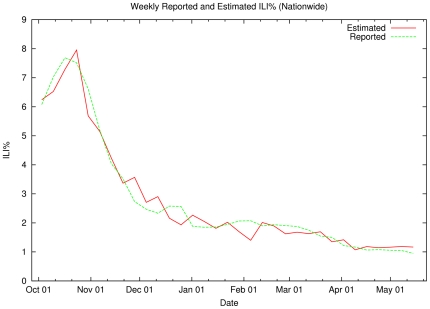
Weekly Reported and Estimated ILI% (Nationwide). The green line shows the CDC's measured ILI% for the 33-week period starting in Week 40 (October 2009) through Week 20 (May 2010). The red line shows the output of a leaving-one-out cross-validation test of our SVM-based estimator. Each estimated datapoint is produced by applying a model to the specified week of tweets after training on the other 32 weeks of data and their respective CDC ILI% values.

In contrast to the descriptive results just described, we next focus on making quantitative estimates of ILI values based on the Twitter stream using support-vector regression. Weekly ILI values were estimated using a model trained on the roughly 1 million influenza-related tweets from the second data set (October 1, 2009 through May 20, 2010) that were unambiguously tagged with US locations, using CDC-reported ILI values across the entire United States as the objective. To verify the accuracy of our method, we used a standard leaving-one-out cross-validation methodology [17], training on 32 times on each 31 week subset of the training data and testing on the remaining week. Figure 9 compares the 32 estimated (red line) ILI values obtained with target ILI values reported by the CDC (green line). These estimates are point estimates, which do not reflect temporal aspects of the data. Even so, the estimates of national ILI values produced by the system are fairly accurate, with an average error of 0.28% (min = 0.04%, max = 0.93%) and a standard deviation of 0.23%.

### Using Twitter to Make Real-Time Estimates of Regional Weekly ILI Levels

We next move beyond estimating national ILI levels to making real-time estimates of ILI activity in a single CDC region. Real-time estimates constitute an important tool for public health practitioners, since CDC-reported data are generally only available one to two weeks after the fact.

Using support vector regression, we fit geolocated tweets to CDC region ILI readings from nine of the ten CDC regions to construct a model. We then use the model to estimate ILI values for the remaining CDC region (Region 2, New Jersey and New York). Since many tweets lacked geographic information, this model was trained and tested on significantly less data (905,497 tweets for which we could accurately infer the US state of origin, less 90,000 of these belonging to Region 2); the remaining tweets were excluded from this analysis.

**Figure 10:**
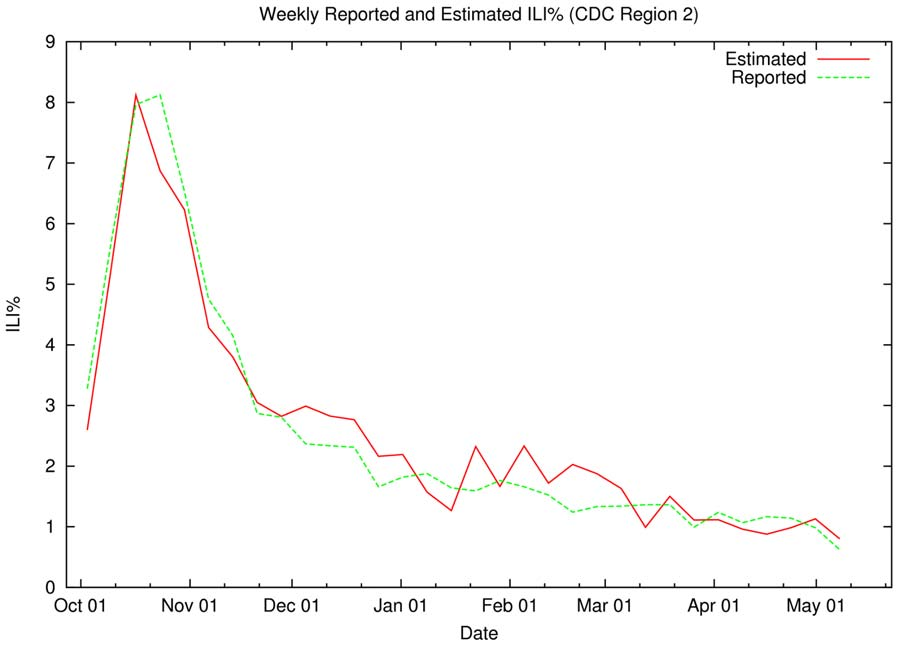
Weekly Reported and Estimated ILI% (CDC Region 2). The green line shows the CDC's measured ILI% for Region 2 (New Jersey/New York) for the 33-week period starting in Week 40 (October 2009) through Week 20 (May 2010). The red line shows the output of our SVM-based estimator when applied to Region 2 tweet data. The estimator is first trained on all data from outside Region 2 and their respective region's CDC ILI% values.

Figure 10 compares the predicted Region 2 weekly ILI values (red line) with the ones reported by the CDC (green line). Note that our regional model still approximates the epidemic curve as reported by ILI data, although this estimate -- based on significantly fewer tweets -- is somewhat less precise than the national weekly ILI model with an average error of 0.37% (min = 0.01%, max = 1.25%) and a standard deviation of 0.26%.

## Discussion

Our results demonstrate that Twitter traffic can be used not only descriptively, i.e., to track users' interest and concerns related to H1N1 influenza, but also to estimate disease activity in real time, i.e., 1–2 weeks faster than current practice allows.

From a descriptive perspective, since no comparable data (e.g., survey results) are available, it is not possible to validate our results. But the trends observed are prima facie reasonable and quite consistent with expectations. For example, Twitter users' initial interest in antiviral drugs such as oseltamivir dropped at about the same time as official disease reports indicated most cases were relatively mild in nature, despite the fact that overall the number of cases was still increasing. Also, interest in hand hygiene and face masks seemed to be timed with public health messages from the CDC about the outbreak in early May. Interestingly, in October of 2009, concern regarding shortages did not appear nor did interest in rare side effects, perhaps because they did not occur in any widespread fashion. Here, absence of a sustained detectable signal may indicate an apathetic public, or may simply indicate a lack of information in the media. In either case, our work proposes a mechanism to capture these concerns in real time, pending future studies to confirm our results using appropriate techniques for analyzing autocorrelated data.

Influenza reoccurs each season in regular cycles, but the geographic location, timing, and size of each outbreak vary, complicating efforts to produce reliable and timely estimates of influenza activity using traditional time series models. Indeed, epidemics are the most difficult to anticipate and model [18]. The literature provides several examples of “syndromic approaches” to anticipating or forecasting ILI, including analyses of telephone triage calls [19], purchases of over-the-counter medications for respiratory diseases [20]–[23], and school absenteeism [24]. While these efforts can yield information about future influenza activity days to weeks in advance of traditional sources (e.g., ILI surveillance), it is difficult to compare these approaches, because different geographic regions were studied and different statistical approaches were used [25].

Using actual tweet contents, which often reflected the user's own level of disease and discomfort (i.e., users were tweeting about their symptoms and body temperature), we devised an estimation method based on well-understood machine learning methods. The accuracy of the resulting real-time ILI estimates clearly demonstrates that the subset of tweets identified and used in our models contains information closely associated with disease activity. Our results show that we were able to establish a distinct relationship between Twitter data and the epidemic curve of the 2009 H1N1 outbreak, both at a national level and within geographic regions.

Our Twitter-based model, in contrast to other approaches [26], does not attempt to forecast influenza activity, but instead to provide real-time estimates. Yet because our results are available “live” (i.e., as soon as the data are captured), our estimates are available sooner than traditional public health reports, which tend to lag ILI activity by 1–2 weeks.

Although, in theory, it is possible to gather diagnosis-level data in near-real time from emergency department visits [27]–[29], doing so at a national level would require fusing, at considerable expense, data sources from different geographic areas and multiple firms (in the case of pharmacy data or billing data): a considerable data management burden. In contrast, like search query data, Twitter data are easily and efficiently collected, and processed automatically in real time. And while search-term data related to influenza is more available than in the past to investigators outside search engine companies, we think that our Twitter-based approach provides some unique advantages. First, the Twitter data provide more contextual information than a corpus of search queries (i.e., lists of key words), so that they can be used to investigate more than just disease activity. Contextual cues also enable the retrospective study of ancillary issues, such as treatment side effects or potential medication shortages. For example, in this study, we investigated perceptions regarding pregnancy and influenza in direct response to a specific question from a state epidemiologist who was concerned that women might avoid the new H1N1 vaccine because of pregnancy-related concerns. It is important for public health officials to know about such opinions, beliefs, and perceptions as they develop, so as to craft more effective communication strategies. Second, Cooper et al. [30] found that daily variations of search frequency in search query data regarding cancer were heavily influenced by news reports, making search query data a necessarily “noisy” marker for actual disease activity. Because the entire tweet is available, this is less of a problem for Twitter-based analysis using the support-vector regression method espoused here, since terms will emerge during model fitting to ensure noisy tweets are excluded. Similar data-mining approaches could also be applied to search data, but require access to more context and state information (e.g., search histories rather than unlinked individual queries) than is generally made available to outside investigators by search-engine firms. This is largely because releasing fine-grained search data raises significant privacy issues, especially if it can be linked to individuals across multiple searches. In contrast, all of the Twitter data used here is placed in the public domain by the issuing user who chooses to broadcast his or her tweets to the world at large: indeed, Twitter and the Library of Congress have future plans to make every public tweet ever issued available to any interested party.

Despite these promising results, there are several limitations to our study. First, the use of Twitter is neither uniform across time or geography. Mondays are usually the busiest for Twitter traffic, while the fewest tweets are issued on Sundays; also, people in California and New York produce far more tweets per person than those in the Midwestern states (or, for that matter, in Europe). When and where tweets are less frequent (or where only a subset of tweets contain geographic information), the performance of our model may suffer. The difference in accuracy at a national level and regional level observed in the Results could, in part, be explained by this lack of data. While the national model used, on average, 120,000 weekly tweets to make its weekly predictions, the regional one had only 3,000. A second limitation is that we only had one year of sampled data. More seasons, especially non-pandemic seasons, should help improve the accuracy of our ILI estimates, as would more complete access to the Twitter stream. Third, the demographic of Twitter users do not represent the general population, and in fact, the exact demographics of the Twitter population, especially the Twitter population that would tweet about health related concerns, is unknown and not easy to estimate. Finally, we need to determine how accurately Twitter can estimate other population-based measures of influenza activity.

If future results are consistent with our findings, Twitter-based surveillance efforts like ours and similar efforts underway in two European research groups [31], [32] may provide an important and cost-effective supplement to traditional disease-surveillance systems, especially in areas of the United States where tweet density is high. We propose that Twitter data can also be used as a proxy measure of the effectiveness of pubic health messaging or public health campaigns. Our ability to detect trends and confirm observations from traditional surveillance approaches make this new form of surveillance a promising area of research at the interface between computer science, epidemiology, and medicine.
